# Identification of Lynch Syndrome Carriers among Patients with Small Bowel Adenocarcinoma

**DOI:** 10.3390/cancers13246378

**Published:** 2021-12-20

**Authors:** Ariadna Sánchez, Luis Bujanda, Miriam Cuatrecasas, Alex Bofill, Cristina Alvarez-Urturi, Goretti Hernandez, Lara Aguilera, Sabela Carballal, Joan Llach, Cristina Herrera-Pariente, Mar Iglesias, Liseth Rivero-Sánchez, Gerhard Jung, Lorena Moreno, Teresa Ocaña, Carolina Bayarri, Maria Pellise, Antoni Castells, Sergi Castellví-Bel, Francesc Balaguer, Leticia Moreira

**Affiliations:** 1 Department of Gastroenterology, Hospital Clínic Barcelona, Centro de Investigación Biomédica en Red en Enfermedades Hepáticas y Digestivas (CIBEREHD), IDIBAPS (Institut d’Investigacions Biomèdiques August Pi i Sunyer), University of Barcelona, 08036 Barcelona, Spain; asanchezg@clinic.cat (A.S.); BOFILL@clinic.cat (A.B.); carballal@clinic.cat (S.C.); JLLACHR@clinic.cat (J.L.); cristina.herrera@ciberehd.org (C.H.-P.); LRIVERO@clinic.cat (L.R.-S.); JUNG@clinic.cat (G.J.); LOMORENO@clinic.cat (L.M.); MOCANA@clinic.cat (T.O.); cbayarri@clinic.cat (C.B.); mpellise@clinic.cat (M.P.); CASTELLS@clinic.cat (A.C.); SBEL@clinic.cat (S.C.-B.); fprunes@clinic.cat (F.B.); 2 Department of Gastroenterology, Biodonostia Health Research Institute, Centro de Investigación Biomédica en Red en Enfermedades Hepáticas y Digestivas (CIBEREHD), Universidad del País Vasco (UPV/EHU), 20014 San Sebastián, Spain; luis.bujanda@osakidetza.net; 3 Department of Pathology, Hospital Clínic Barcelona, Centro de Investigación Biomédica en Red en Enfermedades Hepáticas y Digestivas (CIBEREHD), IDIBAPS (Institut d’Investigacions Biomèdiques August Pi i Sunyer), University of Barcelona, 08036 Barcelona, Spain; mcuatrec@clinic.cat; 4 Department of Gastroenterology, IMIM (Hospital del Mar Medical Research Institute), Barcelona Hospital del Mar, 08003 Barcelona, Spain; 98907@hospitaldelmar.cat; 5 Department of Gastroenterology, Hospital Universitario de Canarias, 38320 Tenerife, Spain; cghmesa@gmail.com; 6 Department of Gastroenterology, Vall d’Hebron Research Institute, 08035 Barcelona, Spain; laraaguilera88@gmail.com; 7 Department of Pathology, IMIM (Hospital del Mar Medical Research Institute), Barcelona Hospital del Mar, 08003 Barcelona, Spain; miglesiascoma@psmar.cat

**Keywords:** Lynch syndrome, small bowel adenocarcinoma, hereditary cancer

## Abstract

**Simple Summary:**

Small bowel adenocarcinoma (SBA) is associated with Lynch syndrome (LS). This is the first study to evaluate the identification of LS patients based on mismatch repair deficiency (MMRd) tumor among SBA. The authors found a 21.3% prevalence of MMRd tumors and a 10.1% prevalence of LS. A germline mutation was identified in 60% of patients with a MMRd tumor. This data suggests that universal tumor MMR testing among SBA patients should be implemented for the identification of LS.

**Abstract:**

Background: Small bowel adenocarcinoma (SBA) is a rare disease which can be associated with Lynch syndrome (LS). LS tumors are characterized by the presence of microsatellite instability (MSI) and/or the loss of mismatch repair (MMR) protein expression. In SBA, the frequency of MMR deficient (MMRd) tumors varies from 5% to 35%. This study aims to describe the prevalence of LS carriers among patients with MMRd small bowel adenocarcinomas. Methods: A multicenter retrospective study with identification and MMR testing of all consecutive SBA between 2004 and 2020 in a multicenter Spanish study. Demographical data, tumor characteristics, follow-up and survival information were collected. Germline testing was driven by identification of MMRd tumors. Results: A total of 94 individuals diagnosed with SBA were recruited. We observed 20 (21.3%) MMRd tumors. In 9/15 (60%) patients with MMRd tumors, a pathogenic variant was identified (three MLH1, four MSH2, one MSH6 and one PMS2). Accordingly, the prevalence of LS among all SBA cases was 10.1%. Conclusions: More than one-fifth of SBA display MMRd and in more than a half is due to LS. Our data supports the implementation of universal MMR tumor testing among SBA for the identification of LS families.

## 1. Introduction

Small bowel adenocarcinoma (SBA) is a rare disease [1]. Although the small bowel represents 90% of the surface area from the gastrointestinal tract, these tumors represent less than 5% of all malignant gastrointestinal neoplasms [2], with a low lifetime risk (0.3%) in the general population. Symptoms are non-specific and generally appear at advanced stages. This late presentation delays diagnoses and results in poor prognoses, with a five-year overall survival of less than 30% [3]. Certain medical conditions have been described as risk factors for SBA, including celiac disease, Crohn’s disease and some hereditary cancer syndromes (i.e., familial adenomatous polyposis (FAP), Peutz-Jeghers syndrome and Lynch syndrome (LS)) [4,5,6,7].

LS is caused by a germline pathogenic variant in a DNA mismatch repair (MMR) gene (MLH1, MSH2, MSH6, PMS2 and 3′ end of EPCAM) and is characterized by an increased risk of cancer, especially colorectal and endometrial cancer [8,9]. LS tumors are characterized by the presence of microsatellite instability (MSI) and/or the loss of MMR protein expression, both considered hallmarks of this disorder [10]. In LS patients, the SBA lifetime cumulative risk rises to 4.2% [11,12].

In SBA, the frequency of MMR deficient (MMRd) tumors varies from 5% to 35% and it is an independent good prognostic factor [13,14,15,16,17]. However, no comprehensive studies have been conducted to identify LS from SBA tumors with altered MMR protein expression.

Identifying LS carriers is of special interest as these patients and their relatives could benefit by undergoing cancer preventive strategies that reduce their cancer incidence and mortality. This study aims to identify patients with LS among those diagnosed with SBA.

## 2. Materials and Methods

### 2.1. Patients and Study Design

From 2004 to 2020, all individuals diagnosed with a small bowel adenocarcinoma (SBA) at four Spanish tertiary hospitals were retrospectively recruited. Clinical and demographic data including age, gender and previous gastrointestinal disease associated with an increased risk of SBA (celiac disease, inflammatory bowel disease and cancer hereditary syndromes), as well as personal and family history of cancer, were registered. Tumor characteristics were reported, including the location, diagnostic stage (TNM), histologic features and grade of differentiation. The type of treatment, follow-up and survival information were also collected. The study was approved by the Hospital Clinic de Barcelona Research Ethics Committee (reference: 2015/0864) and informed consent from individual participants was required.

All tumors underwent mismatch repair (MMR) deficiency testing by immunostaining, including the evaluation of MLH1, MSH2, MSH6 and PMS2 protein expression as previously described [18]. Germline MMR mutational analysis was driven by identification of tumor MMR deficiency. MMR germline genetic testing was performed on germline DNA isolated from peripheral blood leukocytes by both multiple ligation probe amplification analysis and direct sequencing if the patient was alive. When peripheral blood was not available, DNA was extracted from paraffin non-tumoral intestinal tissue. In most patients, a single gene analysis was performed based on the specific loss of protein expression; however, in a few cases, multigene testing was performed through a commercial panel (Trusight Cancer v1, Illumina Inc., San Diego, CA, USA) involving the most frequent hereditary cancer-related genes. LS was diagnosed if a germline pathogenic variant (class 4 and 5 according to InSiGHT classification guidelines) [19] in one of the mismatch repair genes (MLH1, MSH2, MSH6, PMS2 and 3′ end EPCAM deletions) was confirmed [19].

### 2.2. Statistical Analysis

A descriptive analysis was performed. Categorical variables were summarized by proportions and quantitative continuous variables by median and interquartile range (IQR) for non-normally distributed variables, and mean and standard deviation (SD) for normally distributed variables. When information was missing, the denominator accounted for patients with available data. Comparisons between categorical data were performed using Fisher’s exact test. For numerical data, comparisons were performed with the Student’s t-test for parametric and the Mann–Whitney U test for nonparametric data. Overall survival curves were calculated using the Kaplan–Meier method and compared with log-rank test. All calculations were performed with the 22.0 SPSS software package (IBM SPSS Statistics for Window, Version 22.0. Armonk, NY, USA). All tests were two-sided and a *p*-value of less than 0.05 was considered statistically significant.

## 3. Results

### 3.1. General Characteristics

A total of 94 patients with SBA were diagnosed between 2004 and 2020. Demographic and clinical characteristics of the entire cohort are detailed in Table 1.

The median age at SBA diagnosis was 65.5 (IQR 53.75–75.25) years old and 52 (55.3%) patients were male. SBA risk factors were identified: two (2.1%) patients had personal history of celiac disease, five (5.3%) had inflammatory bowel disease (four Crohn’s disease and one ulcerative colitis) and one (1.1%) had familial adenomatous polyposis syndrome with a known pathogenic mutation on the *APC* gene. No other hereditary syndrome was previously known. Twenty-seven (28.7%) patients presented with other metachronous cancers. A family history of cancer was reported in 36 (38.3%) patients. Six cases met clinical diagnostic criteria for LS (four cases fulfilled Amsterdam II criteria and two revised Bethesda criteria) but LS was not suspected prior to the diagnosis of SBA.

### 3.2. Tumor Mismatch Repair Analysis

**Figure 1:**
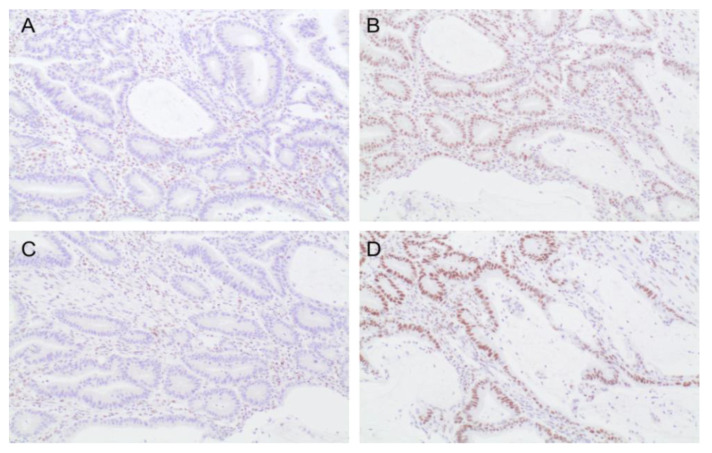
Small bowel adenocarcinoma mismatch repair (MMR) testing by immunostaining (200×) showing: (**A**) MSH1 deficient protein, (**B**) MSH2 proficient protein, (**C**) MSH6 proficient protein and (**D**) PMS2 deficient protein.

MMR tumor testing by immunohistochemistry was performed for the 94 tumors (Table 1). We identified 20 (21.3%) MMRd tumors (Figure 1). Seven (35%) tumors presented a loss of MLH1 and PSM2 protein expression, eight (8.5%) showed a loss of expression of MSH2 and MSH6, two (10%) had an isolated loss of MLH1, one (5%) had an isolated loss of MSH2 and two (10%) had an isolated loss of PMS2. For patients with MMRd tumors, the median age at diagnosis was 58 (IQR 44.5–68.75) years. One patient with a tumor with loss of expression of MLH1 and PMS2 had celiac disease; none of the others had SBA-related diseases. The majority of tumors were proximal: 10 (50%) in the duodenum, nine (45%) in the jejunum and only one (5%) in the ileum. Most MMRd tumors (11/20) were diagnosed in advanced stages (10 (50%) III, 1 (5%) IV).

**Figure 2:**
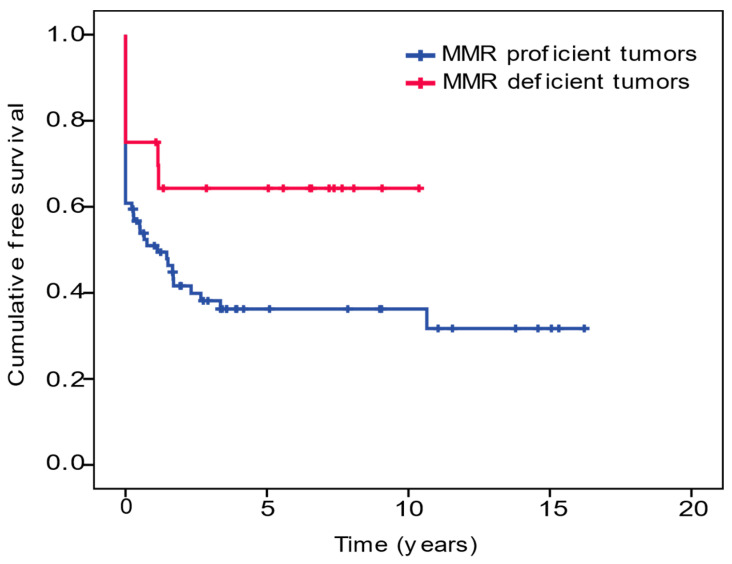
Kaplan-Meyer curves comparing the small-bowel-adenocarcinoma-free survival rate of individuals with a mismatch repair (MMR) proficient and MMR deficient tumors. MMR deficient tumors have a higher mean cancer-free survival: 6.79 (4.66–8.93) years compared to 5.96 (4.18–7.75) years in MMR proficient tumors (*p* = 0.048).

In comparison with patients with MMR proficient tumors, those with MMRd tumors were significantly younger (median age 58 years vs. 68.5 years, respectively, *p* = 0.047) and predominantly located proximally (duodenum and jejunum 19 (95%) vs. 50 (67.6%), *p* = 0.020). There was no difference in either gender, stage at diagnosis or histological grade. However, MMR deficient tumors had a lower cancer mortality (25% vs. 52.7%, respectively, *p* = 0.042), with a significantly higher cancer-free survival (6.79 (95% confident interval (CI) 4.66–8.93) vs. 5.96 (95% CI 4.18–7.75), *p* = 0.048) and five-year overall survival (55% vs. 20.3%, *p* = 0.04) (Table 1 and Figure 2).

### 3.3. MMR Germline Genetic Analysis

We performed a germline genetic study in 15/20 patients with MMRd tumors. In the remaining 5/20 cases, we extracted DNA from formalin-fixed paraffin-embedded non-tumoral intestinal tissue, but the poor condition of the sample prevented the performing of a germline study.

In 9/15 (60%) patients, a pathogenic germline genetic variant was found, leading to the diagnosis of LS: three (20%) in *MLH1*, four (26.7%) in *MSH2*, one (6.7%) in *MSH6* and one (6.7%) in *PMS2* (Table 2).

Among the nine patients with LS, seven (77%) had personal or family history of cancer. Four (44%) individuals had a personal history of colorectal cancer under 50 years old fulfilling the revised Bethesda criteria, while one patient also fulfilled the Amsterdam II criteria. In the other three cases, there was a family history of LS-related tumors but without fulfilling the clinical diagnostic criteria for LS (Table 2).

The median age at SBA diagnosis was 51 (IQR 43–69) years. All tumors had a proximal location: four (44.5%) at the duodenum and five (55.6%) at the jejunum. Four (44.5%) patients were diagnosed in a stage II and five (55.6%) in a stage III. All cases were surgically treated but one tumor was unresectable and the patient died. A second patient died of prostate cancer progression.

## 4. Discussion

This study evaluates the identification of LS patients based on tumor MMR analysis among patients with SBA. Our results show a 21.3% (20/95) prevalence of MMRd tumors and a 10.1% (9/89) prevalence of LS among all SBA cases. Of patients with a MMRd tumor, a germline mutation was identified in 60% of cases.

LS is one of the most common causes of hereditary cancer [20]. These families can benefit from preventive strategies to reduce the incidence and mortality of cancer. In colorectal cancer, universal screening strategies are recommended to improve the diagnosis of LS by performing tumor MMR testing in all cases [8]. However, LS continues to be an underdiagnosed syndrome and our results support this fact. Seven patients diagnosed with a germline mutation had a personal and/or family history of colorectal cancer at early ages and fulfilled the diagnostic criteria to suspect this syndrome. However, these families were not tested for LS before the diagnosis of SBA. Accordingly, universal testing with MMR in all SBA should be recommended.

Moreover, although the incidence of SBA is low (1%) [1] in LS when compared to colorectal cancer (up to 45% lifetime risk) [21], Moreira et al. showed a 13.8% (1386/10019) of MMR deficiency colorectal tumors, with only 20% (289/1386) of them diagnosed with LS [8]. Our results show a better performance of a universal MMR testing strategy in SBA, as more than 20% were MMR deficient and up to 60% were diagnosed with a pathogenic MMR germline mutation.

On the other hand, in LS asymptomatic patients, international guidelines do not recommend systematic surveillance of the small bowel [22]. However, some studies have suggested the use of endoscopic capsule or computed tomography enteroclysis as surveillance techniques, but the low rate of SBA detection has not justified its benefit [12,23,24]. Gastroscopy with a careful duodenal exploration has also been evaluated for the prevention of both gastric and SBA, and the proximal location of SBA in LS supported by our results encourages the performance of prospective studies to evaluate its convenience.

We found significant differences when comparing MMR proficient and deficient tumors. Firstly, MMR deficient tumors are diagnosed at a younger age and at a proximal location. Secondly, although no differences were found on stage and treatment, we found a higher curative intention rate and a higher cancer-free survival. These results support the use of a universal MMR study as an independent factor for better prognosis [13,14].

Moreover, MMRd tumors can express programmed death ligand 1 (PD-L1) and its used as a biomarker to identify immunotherapy-responsive patients [25]. In SBA, the phase II KEYNOTE-158 showed a clinical benefit of anti-PD-1 therapy with pembrolizumab among patients with a histologically confirmed advanced–unresectable and/or metastatic MMRd SBA [26]. The detection of MMR tumors has a clinical implication on treatment decision, reinforcing the importance of universal MMR testing in SBA.

We are aware of some limitations of the study, mainly due to the retrospective design. Firstly, genetic testing was not performed on all patients, although LS with MMR proficient tumors is expected to be exceptional. We must not dismiss that with next-generation gene sequencing panels; a direct analysis of patients with SBA could be also a good strategy to identify LS patients, if accessible. Secondly, the low prevalence of SBA has led to a small number of patients, although we could prove a statistical difference existed between the groups.

## 5. Conclusions

SBA is a rare disease with a poor prognosis. Identifying MMR deficient tumors can predict a better prognosis and identify immunotherapy-responsive patients having immediate clinical implications. Our data shows that 20% of SBA patients display MMRd and more than a half are due to LS MMR germline mutations.

Diagnosis of patients with LS is crucial to identifying pre-symptomatic relatives at risk and to establish preventive measures to decrease morbidity and mortality per cancer.

In this low-prevalence disease, we encourage universal tumor MMR testing implementation to improve treatment decisions and to increase the identification of LS families.

## Figures and Tables

**Table 1:** Patient and tumor characteristics compared between MMR tumor profile.

Characteristics	MMR Proficient Tumor (*n* = 74)	MMR Deficient Tumor (*n* = 20)	*p* Value	Total Patients (*n* = 94)
**Male sex**, *n* (%)	44 (59.5)	12 (60)	0.136	52 (55.3)
Clinical diagnostic criteria for LS *, *n* (%)	2 (2.7)	4 (20)	0.018	6 (6.4)
**Median age at diagnosis**, y (IQR)	68.5 (54.8–77)	58 (44.5–69)	0.047	65.5 (53.8–75.3)
**Stage**			1	
I–II, *n* (%)	30 (45.5)	9 (45)		39 (45.3)
III–IV, *n* (%)	36 (54.5)	11 (55)		47 (54.7)
**Histological grade**			0.915	
G1, *n* (%)	18 (27.3)	5 (25)		23 (26.7)
G2, *n* (%)	25 (37.9)	7 (35)		32 (37.2)
G3, *n* (%)	23 (34.8)	8 (40)		31 (36)
**Location**			0.020	
Duodenum, *n* (%)	31 (42.5)	10 (50)		41 (44.1)
Jejunum, *n* (%)	19 (26)	9 (45)		28 (30.1)
Ileum, *n* (%)	23 (31.5)	1 (5)		24 (25.8)
**Surgical Intervention**, *n* (%)	65 (87.8)	20 (100)	0.197	65 (69.5)
**With curative intention**, *n* (%)	47 (63.5)	18 (90)	0.028	
**Chemotherapy**, *n* (%)	36 (48.6)	11 (55)	0.802	47 (50)
**SBAmortality**, *n* (%)	39 (52.7)	5 (25)	0.042	44 (46.8)
**Median ageSBA mortality,** y (IQR)	72 (58–80)	60 (58.5–85.5)	0.956	71.5 (58.25–80)
**Five-year overall survival** (%)	15 (20.3)	11 (55)	0.004	26 (27.7)
**Mean overall survival,** y (95% CI)	7.121 (5.20–9.04)	6.57 (4.84–8.30)	0.073	7.286 (5.69–8.88)
**Five-year SBA-free survival** (%)	12 (16.2)	10 (50)	0.005	22 (23.4)
**Mean SBA-free survival,** y (95% CI)	5.96 (4.18–7.75)	6.79 (4.66–8.93)	0.048	6.86 (5.21–8.51)

* Amsterdam and or Bethesda criteria. *n*, number; MMR, mismatch repair system; LS, Lynch syndrome; y, years; SBA, small bowel adenocarcinoma.

**Table 2:** Patients and tumor characteristics from MMR deficient tumors.

Sex	SBA Risk Factors	Family History of Cancer	Metachronous Neoplasm	Age at Diagnosis (y)	Location	TNM	Stage	Deficient MMR Proteins	Germline MMR Study	Evolution
Male	No	Two 1st-degree relatives: bladder 40 y-o and pancreatic cancer 38 y-o	CRC 43 y-o	51	Jejunum	T3N1M0	III	MLH1/PMS2	*MLH1* (c.1644 C>G; p. Tyr548Ter)	Alive
Female	No	1st-degree relative CRC 33 y-o; 3rd-degree relative gastric cancer 45 y-o	No	67	Jejunum	T3N0M0	II	MLH1/PMS2	*MLH1* (c.350C>T; p. Thr117Met)	Alive
Female	No	No	CRC 49 y-o	69	Duodenum	T3N0M0	II	MLH1/PMS2	*MLH1* (c.306+5G>A)	Alive
Male	No	Two 1st-degree relatives CRC: 23 and 50 y-o	CRC 41 y-o	42	Jejunum	T4N1M0	III	MSH2/MSH6	*MSH2* (c.1861C>T; p. Arg621Ter)	Alive
Male	No	No	No	44	Duodenum	T4N1M0	III	MSH2/MSH6	*MSH2* (c.927dupAG)	Dead
Male	No	Amsterdam II Criteria: >3 relatives with CRC and/or other LS spectrum cancers	CRC 32 y-o, ureter cancer 44 y-o prostate cancer 65 y-o	69	Duodenum	T3N1M0	III	MSH2/MSH6	*MSH2* (c.1387-?_661+?del)	Dead
Male	No	1st-degree relative CRC 67 y-o	No	46	Ileum	T4N1M0	III	MSH2/MSH6	*MSH2* (c. 842C>G; p. Ser281Ter)	Alive
Female	No	1st-degree relative CRC 60 y-o	Intestinal lymphoma	82	Jejunum	T3N0M0	II	MSH2/MSH6	*MSH6* (c.2188dupT; p. Tyr730LeufsTer26)	Alive
Female	No	1st-degree relative bladder cancer 66 y-o; 2nd-degree relative ureter cancer 67a	No	39	Jejunum	T3N0M0	II	PMS2	*PMS2* (c.1831dup; p. Ile611fs)	Alive
Female	Celiac Disease	Four 2nd-degree relatives CRC > 85 y-o	No	60	Jejunum	T3N0M0	II	MLH1/PMS2	Negative	Alive
Female	No	No	No	71	Jejunum	T3N0M0	II	MLH1	Negative	Alive
Female	No	1st-degree relative endometrium 63 y-o	Follicular lymphoma	37	Duodenum	T3N0M0	II	MLH1/PMS2	Negative	Alive
Male	No	Two 1st-degree relatives: CRC 87 y-o, laryngeal cancer 65 y-o	No	64	Duodenum	T3N2M0	III	MLH1/PMS2	Negative	Alive
Female	No	No	No	57	Duodenum	T4N1M1	IV	MSH2/MSH6	Negative	Dead
Female	No	Three 2nd-degree relatives: leukemia, ovarian cancer, renal cancer	No	43	Duodenum	T4N0M0	II	MSH2/MSH6	Negative	Alive
Female	No	Relative prostate cancer 62 y-o	No	75	Jejunum	T4N2M0	III	MLH1	NA	Dead
Female	No	No	CRC 42 y-o, Hodgkin lymphoma 55 y-o	59	Duodenum	T3N1M0	III	MLH1/PMS2	NA	Dead
Male	No	Relative prostate cancer	No	90	Jejunum	T3N1M0	III	MSH2	NA	Dead
Male	No	3rd-degree relative hepatic cancer	No	53	Duodenum	T3N1M0	III	MSH2/MSH6	NA	Alive
Female	No	1st-degree relative unknown cancer 54 y-o; two 2nd-degree relatives: gastric and intestinal cancer	No	56	Duodenum	T3N0M0	II	PMS2	NA	Dead

MMR, mismatch repair system; SBA, small bowel adenocarcinoma; y, years; y-o, years old; CRC, colorectal cancer; NA, not available.

## Data Availability

The data that support the findings of this study are available from the corresponding author upon reasonable request.
